# Performance of non‐conventional yeasts in co‐culture with brewers’ yeast for steering ethanol and aroma production

**DOI:** 10.1111/1751-7915.12717

**Published:** 2017-08-18

**Authors:** Irma M. H. van Rijswijck, Judith C. M. Wolkers – Rooijackers, Tjakko Abee, Eddy J. Smid

**Affiliations:** ^1^ Laboratory of Food Microbiology Wageningen University & Research Wageningen Campus PO Box 17 6700 AA Wageningen The Netherlands

## Abstract

Increasing interest in new beer types has stimulated the search for approaches to extend the metabolic variation of brewers’ yeast. Therefore, we tested two approaches using non‐conventional yeast to create a beer with lower ethanol content and a complex aroma bouquet. First, the mono‐culture performance was monitored of 49 wild yeast isolates of *Saccharomyces cerevisiae* (16 strains), *Cyberlindnera fabianii* (9 strains) and *Pichia kudriavzevii* (24 strains). Interestingly, both *C. fabianii* and *P. kudriavzevii* isolates produced relatively more esters compared with *S. cerevisiae* isolates, despite their limited fermentation capacity. Next, one representative strain of each species (Sc131, Cf65 and Pk129) was applied as co‐culture with brewers’ yeast (ratio 1:1). Co‐cultures with Cf65 and Pk129 resulted in a beer with lower alcohol content (3.5, 3.8 compared with 4.2% v/v) and relatively more esters. At higher inoculum ratios of Cf65 over brewers’ yeast, growth inhibition of brewers’ yeast was observed, most likely caused by competition for oxygen between brewers’ yeast and Cf65 resulting in a reduced level of ethanol and altered aroma profiles. With this study, we demonstrate the feasibility of using non‐conventional yeast species in co‐cultivation with traditional brewers’ yeast to tailor aroma profiles as well as the final ethanol content of beer.

## Introduction

The brewing industry traditionally uses *Saccharomyces* species as their workhorse to convert wort into beer. These species are extensively studied, have a long history of use and have the generally recognized as safe (GRAS) status (Bourdichon *et al*., 2012). Nowadays, there is an increasing interest in a broad range of new beer types. To create variation, most research focusses on the preparation of the unfermented wort with different carbon/nitrogen ratios, thereby controlling the supply of precursors for aroma compounds (Lei *et al*., 2012). In addition, variation in fermentation conditions (i.e. temperature, fermentation time) has been used to generate diversity in beers (Kaneda *et al*., 1991; Landaud *et al*., 2001; Saerens *et al*., 2008).

Additional research has been done on strain performance, but remarkably this mostly entails single‐strain performance (Zhang *et al*., 2013; Gallone *et al*., 2016). Often, various approaches such as genome shuffling or adaptive evolution experiments are used to generate new variants of strains with desired traits (Steensels *et al*., 2014a,b; Snoek *et al*., 2015). Strain improvements can be obtained to a certain extent via these approaches. However, the degree of variation that can be achieved within one genus (*Saccharomyces*) is smaller compared with what can be obtained in terms of variation when crossing strains from different genera. Recently, Gamero *et al*. (2016) studied the fermentation capacities of a large collection of non‐conventional yeast. Additionally, Basso *et al*. (2016), Steensels and Verstrepen (2014) and Steensels *et al*. (2015) reviewed the use of various non‐conventional yeasts for wort fermentation using various strategies. They all conclude that non‐conventional yeast harbours features of interest for industrial fermentation processes, but the use of those species has to be explored further.

Strategies to apply these non‐conventional yeast species need to be tailored based on their fermentation capacities, such as sugar utilization and aroma/alcohol formation. If the fermentation characteristics are good, selected non‐conventional yeasts can be applied as single‐strain starter culture. Notably, isolates of non‐conventional yeast with poor fermentation characteristics may also be applied for the introduction of specific traits, for example in co‐cultures with a classical brewers’ yeast with proven fermentation capacities. However, to the best of the authors’ knowledge, the latter approach has only been reported once for beer production in a patent describing a sequential (and co‐culture) inoculation of *Pichia* sp. and brewers’ yeast (Saerens and Swiegers, 2014). For wine production, a range of co‐culture approaches have been developed that are currently applied, for example to de‐acidify wine or to increase the complexity of the aroma bouquet (Kim *et al*., 2008; Erten and Tanguler, 2010; Andorrà *et al*., 2012; Del Monaco *et al*., 2014; Quiros *et al*., 2014; Contreras *et al*., 2015).

In this study, we describe the biodiversity and performance of 49 wild yeast isolates in wort fermentation. These isolates originate from fermented masau fruits (Zimbabwe) and belong to the species *Saccharomyces cerevisiae*,* Cyberlindnera fabianii* and *Pichia kudriavzevii*. From this collection of isolates, one representative for each species was chosen (Sc131, Cf65 and Pk129) for application in co‐culture with traditional brewers’ yeast (*S. cerevisiae*). With this study, we demonstrate the feasibility of using non‐conventional yeast species in co‐cultivation with traditional brewers’ yeast to tailor aroma profiles as well as the final ethanol content of beer.

## Results

### Diversity in wort fermentation performance of non‐conventional yeast isolates

**Figure 1:**
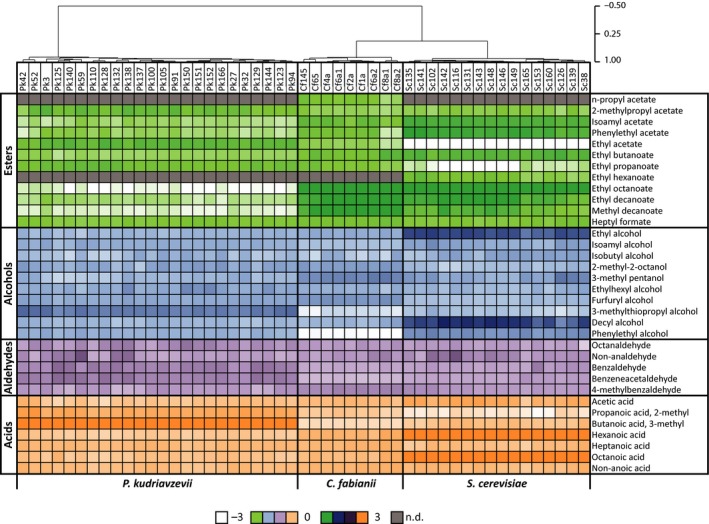
Hierarchical clustered aroma profiles of all 49 wild yeast isolates. A colour gradient is used to indicate the intensity of the compound (0 = median, −3 = below median and 3 = above median); [log_2_(compound/median compound all samples)]. The compounds are grouped as esters (green), alcohols (blue), aldehydes (purple) and acids (orange). Grey means not detected.

The fermentation capacity of 49 wild yeast isolates, belonging to the species *S. cerevisiae* (16 isolates), *C. fabianii* (9 isolates) and *P. kudriavzevii* (24 isolates), was tested on wort after 7 days of incubation at 20°C (Fig. 1). The peak area of the volatile organic compounds (VOCs) was normalized, and hierarchical clustering was applied. A clear clustering is observed per species, indicating that the diversity in VOCs between the three genera is higher than within one species. The relative abundance of the volatile esters compared with the total of volatile alcohols, aldehydes, acids and esters is higher for all *C. fabianii* and *P. kudriavzevii* isolates compared with all *S. cerevisiae* isolates (Table 1). It was demonstrated that all isolates of *C. fabianii* and *P. kudriavzevii* utilized all glucose, but only a limited amount of maltose was used (max 1 g l^−1^). In addition, strains of both species used no maltotriose and produced only approximately 0.6% (v/v) ethanol (Table 1). Glucose was depleted by all *S. cerevisiae* isolates, and all *S. cerevisiae* isolates utilized maltose with an amount varying between 18 and 46 g l^−1^. About 1 g l^−1^ maltotriose was utilized by all *S. cerevisiae* isolates, and only isolates Sc102 and Sc165 utilized more (3 and 6 g l^−1^, respectively). The ethanol formation for the *S. cerevisiae* isolates ranged between 1.9 and 3.7% (v/v).

**Table 1:** Residual wort sugars and ethanol production of all yeast isolates after 7 days of incubation at 20°C (*n* = 4). Sugars are the average in g per l ± SD, ethanol in average % (v/v) ± SD and relative abundance of all volatile alcohols, aldehydes, acids and esters ± standard deviation. Strains Sc131, Cf65 and Pk129 are highlighted in bold

Strain	Residual sugars (g l^−1^)			Ethanol (%v/v)	Relative abundance VOCs per compound group			
	Glucose	Maltose	Maltotriose		Alcohols	Aldehydes	Acids	Esters
Uninoculated wort	10.4 ± 0.6	48.3 ± 2.5	17.3 ± 0.8	0.0 ± 0.0				
Cf145	2.1 ± 0.2	45.1 ± 1.7	15.9 ± 0.7	0.6 ± 0.0	0.53 ± 0.03	0.02 ± 0.00	0.01 ± 0.00	0.44 ± 0.03
Cf1a	1.8 ± 0.1	44.1 ± 0.8	15.3 ± 0.6	0.6 ± 0.0	0.54 ± 0.01	0.02 ± 0.00	0.02 ± 0.00	0.43 ± 0.01
Cf2a	1.9 ± 0.1	45.1 ± 0.6	15.9 ± 0.6	0.6 ± 0.0	0.57 ± 0.01	0.02 ± 0.00	0.02 ± 0.00	0.40 ± 0.01
Cf4a	1.9 ± 0.1	44.5 ± 1.0	15.7 ± 0.7	0.6 ± 0.0	0.55 ± 0.01	0.02 ± 0.00	0.01 ± 0.00	0.42 ± 0.01
Cf65	**2.0 ± 0.3**	**43.7 ± 0.8**	**15.4 ± 0.5**	**0.6 ± 0.0**	**0.56 ± 0.05**	**0.02 ± 0.01**	**0.01 ± 0.00**	**0.41 ± 0.05**
Cf6a1	2.1 ± 0.1	44.9 ± 0.2	16.0 ± 0.4	0.6 ± 0.0	0.56 ± 0.01	0.02 ± 0.00	0.02 ± 0.00	0.41 ± 0.01
Cf6a2	2.1 ± 0.0	44.7 ± 1.1	15.8 ± 0.6	0.6 ± 0.0	0.55 ± 0.01	0.02 ± 0.00	0.01 ± 0.00	0.42 ± 0.01
Cf8a1	2.1 ± 0.4	45.5 ± 1.0	16.0 ± 0.5	0.6 ± 0.0	0.65 ± 0.04	0.02 ± 0.00	0.02 ± 0.00	0.31 ± 0.04
Cf8a2	2.3 ± 0.1	45.9 ± 2.5	16.2 ± 0.8	0.6 ± 0.0	0.62 ± 0.07	0.02 ± 0.00	0.02 ± 0.00	0.35 ± 0.08
Pk100	0.9 ± 0.1	47.0 ± 3.2	17.1 ± 1.1	0.8 ± 0.5	0.46 ± 0.04	0.01 ± 0.00	0.02 ± 0.01	0.50 ± 0.04
Pk105	0.8 ± 0.2	46.9 ± 3.2	16.9 ± 1.0	0.6 ± 0.0	0.45 ± 0.05	0.02 ± 0.00	0.02 ± 0.01	0.52 ± 0.05
Pk110	0.8 ± 0.1	46.5 ± 2.4	16.8 ± 0.9	0.6 ± 0.1	0.51 ± 0.05	0.02 ± 0.00	0.01 ± 0.01	0.46 ± 0.05
Pk123	0.9 ± 0.2	46.8 ± 2.5	17.0 ± 0.7	0.6 ± 0.0	0.48 ± 0.03	0.02 ± 0.00	0.02 ± 0.01	0.49 ± 0.03
Pk125	1.2 ± 0.2	49.2 ± 0.8	18.0 ± 0.2	0.6 ± 0.0	0.43 ± 0.02	0.02 ± 0.00	0.02 ± 0.01	0.53 ± 0.02
Pk128	1.1 ± 0.1	46.7 ± 2.9	16.9 ± 1.3	0.5 ± 0.0	0.40 ± 0.03	0.01 ± 0.00	0.01 ± 0.00	0.57 ± 0.02
Pk129	**0.8 ± 0.1**	**46.0 ± 2.8**	**16.6 ± 1.2**	**0.6 ± 0.1**	**0.48 ± 0.02**	**0.02 ± 0.00**	**0.02 ± 0.01**	**0.48 ± 0.02**
Pk132	0.8 ± 0.1	46.1 ± 2.3	16.9 ± 0.7	0.6 ± 0.0	0.46 ± 0.03	0.02 ± 0.00	0.02 ± 0.00	0.51 ± 0.03
Pk137	0.8 ± 0.1	46.2 ± 2.6	16.9 ± 1.0	0.6 ± 0.0	0.43 ± 0.01	0.01 ± 0.00	0.01 ± 0.00	0.54 ± 0.01
Pk138	0.9 ± 0.1	46.1 ± 2.0	16.7 ± 0.9	0.6 ± 0.1	0.52 ± 0.03	0.02 ± 0.00	0.02 ± 0.01	0.44 ± 0.02
Pk140	1.2 ± 0.2	45.3 ± 1.8	16.6 ± 0.5	0.5 ± 0.0	0.42 ± 0.02	0.02 ± 0.00	0.02 ± 0.01	0.55 ± 0.02
Pk144	0.8 ± 0.1	46.5 ± 2.5	16.9 ± 1.3	0.6 ± 0.0	0.48 ± 0.02	0.02 ± 0.00	0.01 ± 0.01	0.49 ± 0.02
Pk150	0.8 ± 0.1	46.5 ± 2.3	17.1 ± 0.6	0.6 ± 0.0	0.47 ± 0.03	0.01 ± 0.00	0.01 ± 0.01	0.51 ± 0.02
Pk151	0.8 ± 0.1	45.9 ± 1.6	16.6 ± 0.6	0.6 ± 0.0	0.48 ± 0.02	0.01 ± 0.00	0.01 ± 0.01	0.49 ± 0.02
Pk152	0.8 ± 0.0	46.8 ± 3.2	16.9 ± 1.0	0.6 ± 0.0	0.47 ± 0.02	0.01 ± 0.00	0.01 ± 0.00	0.50 ± 0.02
Pk166	0.8 ± 0.1	46.2 ± 2.3	16.7 ± 0.7	0.6 ± 0.0	0.48 ± 0.02	0.02 ± 0.00	0.02 ± 0.01	0.48 ± 0.02
Pk27	0.8 ± 0.1	46.1 ± 2.2	16.8 ± 0.5	0.6 ± 0.0	0.49 ± 0.02	0.01 ± 0.00	0.02 ± 0.01	0.48 ± 0.02
Pk3	0.8 ± 0.1	46.1 ± 1.2	16.6 ± 0.3	0.6 ± 0.0	0.48 ± 0.03	0.01 ± 0.00	0.02 ± 0.01	0.48 ± 0.02
Pk32	0.8 ± 0.1	45.9 ± 1.0	16.6 ± 0.2	0.6 ± 0.0	0.51 ± 0.02	0.02 ± 0.00	0.02 ± 0.01	0.46 ± 0.02
Pk42	0.8 ± 0.0	45.7 ± 1.8	16.3 ± 0.6	0.6 ± 0.0	0.56 ± 0.02	0.02 ± 0.00	0.03 ± 0.01	0.40 ± 0.02
Pk52	0.8 ± 0.0	45.8 ± 2.1	16.8 ± 0.8	0.6 ± 0.0	0.53 ± 0.05	0.02 ± 0.00	0.03 ± 0.01	0.42 ± 0.04
Pk59	0.9 ± 0.2	45.2 ± 1.7	16.2 ± 0.4	0.5 ± 0.1	0.47 ± 0.06	0.02 ± 0.01	0.02 ± 0.01	0.49 ± 0.06
Pk91	0.9 ± 0.1	46.3 ± 2.5	16.9 ± 0.9	0.6 ± 0.0	0.44 ± 0.03	0.01 ± 0.00	0.02 ± 0.01	0.53 ± 0.03
Pk94	0.8 ± 0.1	45.8 ± 2.1	16.6 ± 0.6	0.6 ± 0.0	0.45 ± 0.03	0.02 ± 0.00	0.02 ± 0.01	0.52 ± 0.03
Sc102	0.2 ± 0.0	1.9 ± 0.3	14.1 ± 1.0	3.7 ± 0.1	0.82 ± 0.01	0.01 ± 0.00	0.04 ± 0.00	0.13 ± 0.01
Sc116	0.6 ± 0.1	3.2 ± 0.0	15.9 ± 0.9	3.4 ± 0.1	0.76 ± 0.01	0.01 ± 0.00	0.07 ± 0.00	0.16 ± 0.00
Sc126	0.7 ± 0.0	7.0 ± 2.0	16.6 ± 0.8	3.1 ± 0.1	0.80 ± 0.01	0.01 ± 0.00	0.06 ± 0.00	0.12 ± 0.01
Sc131	**0.6 ± 0.1**	**3.1 ± 0.2**	**16.3 ± 1.1**	**3.4 ± 0.1**	**0.78 ± 0.01**	**0.01 ± 0.00**	**0.07 ± 0.01**	**0.14 ± 0.01**
Sc135	0.2 ± 0.0	4.7 ± 0.1	16.1 ± 0.8	3.4 ± 0.1	0.77 ± 0.02	0.01 ± 0.00	0.05 ± 0.00	0.17 ± 0.01
Sc139	0.3 ± 0.0	19.9 ± 0.6	16.5 ± 0.6	2.4 ± 0.0	0.79 ± 0.00	0.01 ± 0.00	0.07 ± 0.01	0.13 ± 0.01
Sc141	0.2 ± 0.0	4.9 ± 0.5	16.1 ± 0.6	3.4 ± 0.1	0.76 ± 0.01	0.01 ± 0.00	0.04 ± 0.00	0.18 ± 0.01
Sc142	0.7 ± 0.1	3.0 ± 0.1	16.3 ± 0.8	3.5 ± 0.1	0.76 ± 0.01	0.01 ± 0.00	0.07 ± 0.00	0.16 ± 0.01
Sc143	0.7 ± 0.1	3.3 ± 0.1	16.1 ± 0.4	3.4 ± 0.1	0.78 ± 0.01	0.01 ± 0.00	0.07 ± 0.00	0.15 ± 0.01
Sc146	0.6 ± 0.0	3.2 ± 0.1	16.1 ± 0.5	3.4 ± 0.1	0.77 ± 0.02	0.01 ± 0.00	0.07 ± 0.00	0.16 ± 0.02
Sc148	0.6 ± 0.0	3.3 ± 0.1	16.0 ± 0.6	3.4 ± 0.1	0.75 ± 0.01	0.01 ± 0.00	0.08 ± 0.00	0.17 ± 0.01
Sc149	0.7 ± 0.1	3.4 ± 0.4	16.0 ± 0.6	3.4 ± 0.1	0.76 ± 0.01	0.01 ± 0.00	0.07 ± 0.01	0.16 ± 0.01
Sc153	0.3 ± 0.0	20.5 ± 1.2	16.3 ± 0.6	2.4 ± 0.1	0.74 ± 0.02	0.01 ± 0.00	0.08 ± 0.00	0.17 ± 0.01
Sc160	0.3 ± 0.0	17.9 ± 0.4	16.3 ± 0.8	2.5 ± 0.1	0.72 ± 0.01	0.01 ± 0.00	0.09 ± 0.00	0.18 ± 0.01
Sc165	0.3 ± 0.0	30.7 ± 1.2	11.2 ± 0.5	1.9 ± 0.0	0.75 ± 0.01	0.01 ± 0.00	0.05 ± 0.00	0.18 ± 0.01
Sc38	0.2 ± 0.0	21.7 ± 2.8	16.3 ± 0.7	2.2 ± 0.0	0.78 ± 0.01	0.01 ± 0.00	0.07 ± 0.00	0.14 ± 0.01
Blank	10.4 ± 0.6	48.3 ± 2.5	17.3 ± 0.8	0.0 ± 0.0				

These results indicate that it is possible to tailor the final ethanol content and aroma profile by using non‐conventional yeast species, such as *C. fabianii* and *P. kudriavzevii*. It also suggests that the composition of the final fermented product potentially can be influenced using a co‐culture of brewers’ yeast and one of the non‐conventional yeast species. To investigate this concept further, one isolate per species was chosen: *S. cerevisiae* 131 (Sc131), *C. fabianii* 65 (Cf65) and *P. kudriavzevii* 129 (Pk129). As reference, a commercial (Ale) brewers’ yeast was used (*S. cerevisiae*, Lallemand Danstar ‘Nottingham’) to which we will refer to as brewers’ yeast.

### Wort fermentation using co‐cultures

**Figure 2:**
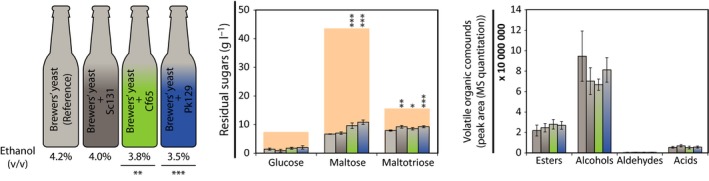
Characteristics of the wort fermented (*t* = end) with a mono‐culture of brewers’ yeast (reference) or a co‐culture of brewers’ yeast with Sc131, Cf65 and Pk129. Top bottles explain the colour code for each variant: brewers’ yeast (light grey), Sc131 (dark grey), Cf65 (green), Pk129 (blue). For the co‐cultures, there is a gradient of the colours indicating the 1:1 ratio of the two strains. The ethanol content (% v/v) is listed below the bottles. Residual sugars are displayed in g l^−1^ (middle), and orange indicates the amount of sugars present at *t* = 0. The sum of MS quantification for all esters, alcohols, aldehydes and acids is displayed on the right. Statistical differences (Student's *t*‐test) compared with the mono‐culture of brewers’ yeast are indicated with stars; *P <* *0.05, **P <* *0.01, *** *P *<* *0.001.

The three representative isolates (Sc131, Cf65 and Pk129) were applied as co‐culture with brewers’ yeast in a ratio 1:1. Various parameters were analysed [i.e. residual sugars, ethanol and volatile organic compounds (VOCs)] after 1 week of primary fermentation and 6 weeks of secondary fermentation (final product). Subsequently, the performance of the co‐cultures was compared with the performance of brewers’ yeast as mono‐culture (Fig. 2, Table 2). Similar results were found for brewers’ yeast in mono‐culture and in co‐culture with Sc131 except for the maltotriose consumption, which was significantly less for the co‐culture with Sc131. Significantly less maltose and maltotriose were consumed by the co‐cultures with Cf65 and Pk129 corresponding to significantly less ethanol formation (3.8 ± 0.1% and 3.5 ± 0.1% (v/v), respectively compared with 4,2 ± 0.1% (v/v) by brewers’ yeast in mono‐culture, Fig. 2, Table 2). Interestingly, no significant difference was found for the volatile esters and alcohols for the co‐culture of brewers’ yeast with Cf65 or Pk129. However, if we compare the ratio of each compound group per fermentation, it is clear that the co‐culture of brewers’ yeast with Cf65 produces relatively more esters compared with the mono‐culture of brewers’ yeast (fraction of 0.28 for the co‐culture with Cf65 and 0.19 for the mono‐culture of brewers’ yeast, Table 2). The complexity of the volatile organic compounds is indicated by the Shannon diversity index. A significantly higher index number was found for the co‐cultures of brewers’ yeast with either Cf65 or Pk129, and this means that composition of the VOCs is more complex for these co‐cultures and that this approach influenced the end‐product.

**Table 2:** Parameters at the end of secondary fermentation of all mono‐ and co‐cultures (*n* = 4)

	Residual sugars (g l^−1^)			Ethanol % (v/v)	CO_2_ produced (mmol)	Relative abundance VOCs per compound group				Shannon index
	Glucose	Maltose	Maltotriose			Esters	Alcohols	Aldehydes	Acids	
Mono‐cultures										
brewers’ yeast	1.4 ± 0.4	6.7 ± 0.1	7.9 ± 0.2	4.19 ± 0.15	0.297 ± 0.005	0.19 ± 0.06	0.76 ± 0.08	0.00 ± 0.00	0.05 ± 0.01	1.72 ± 0.23
Sc131	1.9 ± 0.0*	12.1 ± 1.1*	13.5 ± 0.3*	3.18 ± 0.06*	0.261 ± 0.031	0.19 ± 0.03	0.71 ± 0.05	0.01 ± 0.00	0.09 ± 0.01*	1.84 ± 0.16
Cf65	6.4 ± 0.1*	39.2 ± 1.2*	13.7 ± 0.3*	0.49 ± 0.02*	0.013 ± 0.001*	0.43 ± 0.08*	0.55 ± 0.08*	0.01 ± 0.01	0.01 ± 0.00*	1.86 ± 0.08
Pk129	2.7 ± 0.7*	45.3 ± 1.6*	14.4 ± 0.9*	0.53 ± 0.02*	0.015 ± 0.001*	0.60 ± 0.02*	0.38 ± 0.03*	0.01 ± 0.01	0.01 ± 0.00*	1.38 ± 0.07
Co‐cultures (ratio 1:1)										
brewers’ yeast:Sc131	0.9 ± 0.5	7.0 ± 0.4	9.2 ± 0.5*	4.02 ± 0.15	0.286 ± 0.018	0.24 ± 0.05	0.68 ± 0.05	0.01 ± 0.00	0.07 ± 0.01*	1.97 ± 0.12
brewers’ yeast:Cf65	1.8 ± 0.4	9.6 ± 1.0*	8.6 ± 0.4*	3.80 ± 0.12*	0.273 ± 0.008*	0.28 ± 0.03*	0.66 ± 0.04	0.01 ± 0.00	0.05 ± 0.01	2.05 ± 0.07*
brewers’ yeast:Pk129	2.0 ± 0.6	10.8 ± 0.8*	9.3 ± 0.4*	3.55 ± 0.12*	0.249 ± 0.007*	0.24 ± 0.03	0.71 ± 0.03	0.01 ± 0.00	0.05 ± 0.01	1.89 ± 0.03*
Dose–response (brewers’ yeast: Cf65)										
200:1	1.6 ± 0.4	6.8 ± 0.5	8.1 ± 0.1	4.19 ± 0.20	0.297 ± 0.010	0.18 ± 0.02	0.77 ± 0.02	0.00 ± 0.00	0.05 ± 0.00	1.67 ± 0.13
100:1	1.4 ± 0.3	6.6 ± 0.5	7.9 ± 0.3	4.11 ± 0.18	0.299 ± 0.006	0.20 ± 0.07	0.74 ± 0.08	0.01 ± 0.00	0.05 ± 0.02	1.77 ± 0.31
10:1	1.3 ± 0.7	6.7 ± 0.7	7.9 ± 0.3	4.09 ± 0.21	0.292 ± 0.012	0.22 ± 0.07	0.72 ± 0.08	0.01 ± 0.00	0.05 ± 0.01	1.84 ± 0.17
1:1	1.8 ± 0.4	9.6 ± 1.0*	8.6 ± 0.4*	3.80 ± 0.12*	0.273 ± 0.008*	0.28 ± 0.03*	0.66 ± 0.04	0.01 ± 0.00	0.05 ± 0.01	2.05 ± 0.07*
1:10	1.9 ± 0.4	17.8 ± 0.9*	10.9 ± 0.4*	2.97 ± 0.18*	0.198 ± 0.011*	0.31 ± 0.03*	0.61 ± 0.03*	0.01 ± 0.00	0.07 ± 0.02	2.14 ± 0.08*
1:100	2.2 ± 0.4*	26.6 ± 1.3*	12.3 ± 0.2*	1.99 ± 0.04*	0.118 ± 0.008*	0.36 ± 0.06*	0.54 ± 0.04*	0.01 ± 0.01	0.09 ± 0.02*	2.26 ± 0.18*
1:200	2.4 ± 0.3*	30.9 ± 2.9*	13.0 ± 0.9*	1.75 ± 0.13*	0.097 ± 0.008*	0.35 ± 0.03*	0.55 ± 0.06*	0.01 ± 0.01	0.08 ± 0.03*	2.20 ± 0.25*

The average ± SD of residual sugars (glucose, maltose and maltotriose), ethanol % (v/v), CO_2_ (mmol), relative abundance VOCs per compound group (esters, alcohols, aldehydes and acids) and the Shannon index are listed. Significant difference against brewers’ yeast (bold) is indicated with a star (*P <* *0.05).

Often, cultures that are used for additional functionalities such as aroma formation and not for their fermentative capabilities are added at a higher dose compared with the culture with fermentation capabilities (El Soda *et al*., 2000). Therefore, we tested whether there is a dose–response relationship linked to the addition of Cf65 to brewers’ yeast that affects final metabolite and aroma profiles.

### Dose–response effect of *C. fabianii* in co‐cultivation

**Figure 3:**
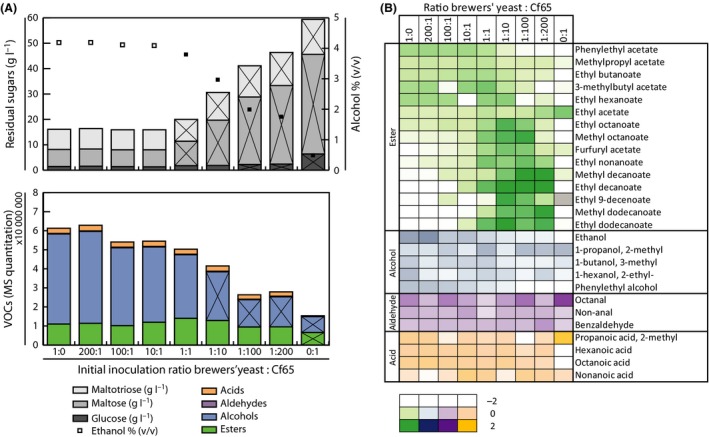
Dose–response effect. Box A, top graph: residual sugars [glucose (dark grey), maltose (mid grey) and maltotriose (light grey)] and ethanol % (v/v, open squares) in the final product. Box A, bottom graph: sum of VOCs per group of compounds [esters (green), alcohols (blue), aldehydes (purple) and acids (orange)]. Significant difference to initial inoculum [only brewers’ yeast (ratio 1:0)] is indicated by black cross in the bar (residual sugars and VOCs) and black closed squares (ethanol) (*P *<* *0.05). Box B: A colour gradient is used to indicate the intensity of the compound among the different inoculation ratios (0 = median, −3 = below median and 3 = above median); [log_2_(compound/median compound all samples)]. The compounds are grouped as esters (green), alcohols (blue), aldehydes (purple) and acids (orange). Grey = not detected.

Various ratios of brewers’ yeast to Cf65 were tested (1:0, 200:1, 100:1, 10:1, 1:1, 1:10, 1:100, 1:200 and 0:1). In the final fermented beverage, a clear dose–response relationship was observed regarding the sugar utilization, ethanol production (% v/v), the formation of VOCs and CO_2_ production (Fig. 3 and Table 2).

Interestingly, a negative correlation was observed between the amount of Cf65 inoculated and sum of all volatile alcohols produced, while a positive correlation was observed for the sum of all volatile esters (up to an initial inoculum ratio of 1:10), and no difference was found for the volatile aldehydes and acids (Fig. 3A, Table 2). This results also in a change in relative abundance per compound group (Table 2), which is very important for the sensorial characteristic of the final product. Fig. 3B displays the dose–response relationship per compound. Phenylethyl acetate, methylpropyl acetate, ethyl butanoate, 3‐methylbutyl acetate, ethyl hexanoate and all volatile alcohols showed a negative correlation towards the increasing initial inoculum ratio of Cf65 over brewers’ yeast. In contrast, a positive correlation towards increasing initial ratios Cf65 was found for ethyl acetate, ethyl octanoate, methyl octanoate, furfuryl acetate, ethyl nonanoate, methyl decanoate, ethyl decanoate, ethyl 9‐decanoate, methyl dodecanoate and ethyl dodecanoate (Fig. 3B). The Shannon diversity index of the VOCs composition was calculated. The index increased with increasing Cf65 amounts and is significantly higher for the initial inoculum ratios (brewers’ yeast: Cf65) 1:1, 1:10, 1:100 and 1:200 compared with the mono‐culture of brewers’ yeast, which indicates a more complex aroma bouquet when Cf65 is most abundant in the initial inoculum (Table 2).

Remarkably, brewers’ yeast does not dominate the fermentation in all tested conditions despite the presence of residual sugars in the end‐product. This indicates that either brewers’ yeast is inhibited by compounds produced by Cf65 (e.g. killer toxins) or that compounds needed for growth of brewers’ yeast are utilized by Cf65. As no evidence was found for the production of inhibitory compounds by Cf65 (Fig. S1), the second option was investigated.

### brewers’ yeast is inhibited due to low oxygen availability

Cf65 efficiently grows in the presence of oxygen displaying higher growth rates than brewers’ yeast, but it cannot grow anaerobically. In aerobic conditions, brewers’ yeast grows on sugars in a fermentative mode (Crabtree‐effect) and it can grow anaerobically but requires traces of molecular oxygen to synthesize ergosterol and unsaturated fatty acids (UFAs). Supplementation of wort with ET80 (ergosterol + Tween 80) ensures that brewers’ yeast grows in the complete absence of molecular oxygen (Longley *et al*., 1978). To test whether growth of brewers’ yeast in co‐culture with Cf65 is inhibited by the lack of molecular oxygen, brewers’ yeast was cultivated in a bioreactor (batch culture) together with Cf65 (inoculation ratio 1:100, with 10^4^ and 10^6^ CFU ml^−1^, respectively) in wort with and without the supplementation of ET80.

**Figure 4:**
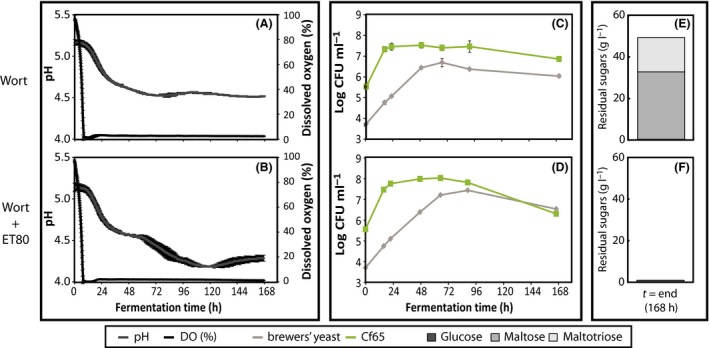
Effect of ET80 supplementation on yeast growth and performance. Wort, either with (B, D and E) or without (A, C and E) ET80, was initially inoculated with brewers’ yeast + Cf65 (ratio 1:10). Dissolved oxygen (black, A and B), pH (dark grey, A and B) and colony‐forming units of brewers’ yeast (grey, C and D) and Cf65 (green, C and D) were followed over time (average ± SD, *n* = 4). The residual sugars [glucose (dark grey), maltose (mid grey) and maltotriose (light grey)] were measured at the end of fermentation (E and F).

The dissolved oxygen decreases in both fermentations from 100% to approximately 0% within 8 h (Fig. 4A and B). During the batch cultivation, the pH drops from 5.2 to 4.5 in 58 h for both fermentations. beyond 58 h, the pH of the culture supplemented with ET80 reaches 4.2 in 115 h, while the non‐supplemented culture remained at pH 4.5 (Fig. 4A and B). In line with these results, we found the plate counts of brewers’ yeast to increase to 7.4 ± 0.04 log CFU ml^−1^ in the culture supplemented with ET80 and to 6.7 ± 0.2 log CFU ml^−1^ in the non‐supplemented wort (Fig. 4C and D). Moreover, only trace amounts of residual sugar were detected after 7 days of fermentation in the culture supplemented with ET80, while significantly more residual sugar was found in the non‐supplemented culture (Fig. 4E and F). These results align with the conclusion that brewers’ yeast is inhibited by oxygen depletion in a co‐culture with Cf65 unless ET80 is added. The observation that no residual sugars are left in the culture supplemented with ET80 confirms that there are no other inhibiting factors for brewers’ yeast (Fig. 4F and Fig. S1).

## Discussion

For wort fermentation, usually a mono‐culture of an in‐house brewers’ yeast is used. This is either *Saccharomyces cerevisiae* (ale‐type yeast) or a natural hybrid of *S. cerevisiae* and *S. eubayanus* (lager‐type yeast) (Hebly *et al*., 2015). Many attempts to screen for or develop strains with diverse characteristics have been made; however, the range of biodiversity is limited when strains are sourced from a single genus (Saerens *et al*., 2010; Steensels *et al*., 2012, 2014a,b; Snoek *et al*., 2015; Gallone *et al*., 2016). To increase the range of diversity, a number of research groups have recently investigated the use of non‐*Saccharomyces* yeast (the so‐called non‐conventional yeast) (Johnson, 2013; Jolly *et al*., 2014; Steensels and Verstrepen, 2014; Steensels *et al*., 2015; Gamero *et al*., 2016). They all conclude that non‐conventional yeast harbour interesting traits for industrial fermentations; however, an approach for their application in wort fermentation remains to be explored. Here, we investigated the use of (non‐)conventional yeast in mono‐cultivation and co‐cultivation with brewers’ yeast (*S. cerevisiae)*. These approaches potentially offer the opportunity to add new product characteristics to fermented beverages.

We first monitored the diversity in aroma production and growth characteristics among 49 wild yeast isolates, belonging to the species *S. cerevisiae*,* C. fabianii* and *P. kudriavzevii* in wort. Both *C. fabianii* and *P. kudriavzevii* are not able to grow in the absence of oxygen and therefore can only grow at early stages of the fermentation. Hence, they mainly utilized glucose, where *S. cerevisiae* strains utilized – as expected ‐ glucose and maltose. The potential benefit of using *C. fabianii* and *P. kudriavzevii* is their ability to produce relatively high levels of esters in comparison with *S. cerevisiae* (Table 1) which is in line with findings by Nyanga *et al*. (2013). Additionally, *C. fabianii* and *P. kudriavzevii* produce not only relatively more esters, but also the balance of the different esters is different compared with *S. cerevisiae* (Fig. 1).

In the brewing industry, it is custom to use pure cultures consisting of a single strain to ferment wort, as breweries tend to recycle their yeast for a few batches. However, in various other fermentation industries (i.e. fermented dairy, wine), it is common practice to add multiple strains to obtain a consistent, more complex aroma bouquet which can be fine‐tuned by varying the dose of each strain (El Soda *et al*., 2000; Ciani *et al*., 2010; Andorrà *et al*., 2012; Spus *et al*., 2017). Recently, Saerens and Swiegers (2014) patented the use of co‐cultivation of *Pichia kluyverii* and brewers’ yeast to produce a beer with enhanced flavour. However, no data on aroma and ethanol formation in the co‐cultivation conditions were supplied, neither is the synergy between the two yeast species described.

In our study, co‐cultivation was performed with one representative strain per species selected based on the performance as mono‐culture: Sc131, Cf65 and Pk129. These strains were co‐cultivated with brewers’ yeast at a ratio of 1:1. Due to the poor fermentation performance of Cf65 and Pk129 in mono‐cultures, brewers’ yeast was expected to dominate the fermentation, as it can perform best under the applied conditions. Interestingly, we found significantly more residual sugars in the co‐cultures (maltotriose for all co‐cultures and maltose for the co‐culture with Cf65 and Pk129). This observation aligns with the detected reduced ethanol production. Interestingly, the relative abundance of volatile esters in the co‐cultivation of Cf65 with brewers’ yeast is significantly higher (Table 2) compared with what is found in the brewers’ yeast mono‐culture. Therefore, we chose to further explore the fermentation process by varying the inoculum ratios of Cf65 over brewers’ yeast.

Interestingly, a clear dose–response relationship was observed. A higher initial dose of Cf65 resulted in more residual sugars, lower ethanol % (v/v), a decrease in volatile alcohols and a increase in relative abundance of volatile esters. This relationship shows that it is possible to steer the end‐product composition by varying the ratios of Cf65 and brewers’ yeast. Esters are the one the most important aroma compounds determining the flavour, due to their low‐odour threshold. Among the ester, the most important compounds are ethyl acetate (solvent‐like aroma), 3‐methylbutyl acetate (banana aroma), methylpropyl acetate (fruity, sweet aroma), phenylethyl acetate (rose, apple, honey aroma), ethyl hexanoate (apple, aniseed aroma) and ethyl octanoate (sour apple aroma) and are either positively or negatively correlated with a higher initial inoculation dose of Cf65 (Fig. 3B; Michel *et al*., 2016). If the concentration of these compounds is too high, it is perceived as an off‐flavour; therefore, the balance between all aroma compounds is of utmost importance.

Additionally, the complexity of the VOCs increases with a higher dose of Cf65 (Shannon diversity index, Table 2). Interestingly, no dose–response relationship was found for the volatile aldehydes and acids. These findings indicate that the sensorial properties can be changed by varying the initial inoculum ratios of Cf65 and brewers’ yeast. However, the VOCs profile does not provide information on the sensorial impact. Therefore, it is recommended for future studies to evaluate product characteristics using a sensorial panel.

The observed dose–response relationship is the result of competition between Cf65 and brewers’ yeast. We found no evidence for the production of compounds by Cf65 which inhibit brewers’ yeast (Fig. S1). Further investigation revealed that fast oxygen depletion by Cf65 inhibits the performance of brewers’ yeast. brewers’ yeast needs oxygen to synthesize ergosterol [12 moles oxygen per mole ergosterol (Rosenfeld and Beauvoit, 2003)] when insufficient amounts are stored intracellularly. Supplementation of wort with ET80 overrules this effect and resulted in complete fermentation of all sugars by brewers’ yeast in the co‐culture. This also indicates that there are no other factors inhibiting the performance of brewers’ yeast in co‐culture with Cf65.

It needs to be stressed that the conditions of preculturing could also affect the outcome of the competition. In our experimental design, brewers’ yeast was pre‐grown in static cultures and it is conceivable that pre‐growth in aerated cultures results in higher intracellular ergosterol levels that may enable completion of the fermentation in the presence of Cf65. Obviously, this is one of the parameters that can be included in future co‐culture experiments.

Understanding the underlying mechanism of the dose–response relationship of Cf65 in co‐culture with brewers’ yeast makes it now possible to apply the same principle for other (aerobic) non‐conventional yeast selected after screening for specific features including novel (combinations of) aroma compounds, such as Pk129 identified in the current study. Additionally, the dose–response relation makes it possible to model the performance of both yeasts to optimize the optimal ratio Cf65 over brewers’ yeast by prediction of the final product characteristics (such as volatile esters an ethanol content). This approach can therefore now be used for product innovation to enhance the aroma bouquet of the final product beyond the capacities of brewers’ yeast alone.

## Materials and methods

### Yeast strains

A total of 49 yeast strains isolated from fermented masau fruit as described by Nyanga *et al*. (2007) and were used in this study: 16 *Saccharomyces cerevisiae* strains (Sc38, Sc102, Sc116, Sc126, Sc131, Sc135, Sc139, Sc141, Sc142, Sc143, Sc146, Sc148, Sc149, Sc153, Sc160, Sc165), 9 *Cyberlindnera fabianii* (formerly named *Pichia fabianii, Hansenula fabianii* and *Lindnera fabianii*) strains (Cf65, Cf145, Cf1a, Cf2a, Cf4a, Cf6a1, Cf6a2, Cf8a1 and Cf8a2) and 24 *Pichia kudriavzevii (*formerly named *Issatchenkia orientalis)* strains (Pk5, Pk27, Pk32, Pk42, Pk52, Pk59, Pk91, Pk94, Pk100, Pk105, Pk110, Pk123, Pk125, Pk128, Pk129, Pk132, Pk137, Pk138, Pk140, Pk144, Pk150, Pk151, Pk152 and Pk166). As a reference strain, a brewers’ yeast (*S. cerevisiae*) from Lallemand Danstar called ‘Nottingham’ was used. All strains were stored at −80°C in 15% (v/v) glycerol.

### Wort preparation

Standardized wort concentrate (Brewferm^®^ Pils Hopped Malt extract) was used to avoid differences between batches of wort. One can (1.5 kg malt extract) was dissolved in 12 L water (12° Brix). The wort was heated for 15 min at 105°C to avoid contamination and kept refrigerated until use.

### Biodiversity of 49 wild isolates on wort (100 ml cultures)

All strains were streaked from glycerol stock onto malt extract agar (MEA, Oxoid Limited, Basingstoke, UK) plates and incubated for 3 days at 20°C. A single colony was inoculated in 15 ml wort and incubated for 22 h at 30°C and 160 rpm. The optical density (OD_600 nm_) of these cultures was measured to calculate the total amount of the inoculum to be used for the fermentation (starting OD_600 nm_ = 0.5, total volume = 100 ml). The flasks were well shaken to introduce oxygen before closing the flasks with a plug and water lock and incubated at 20°C for 7 days (primary fermentation). After primary fermentation, the volatile organic compounds, ethanol formation and sugar utilization were measured.

### Wort fermentation in Schott flasks for co‐culture/dose–response experiment

Yeasts were precultured starting with a streak on MEA plates from glycerol stocks and incubated for 24 h at 30°C. A single colony of each plate was inoculated in 250 ml Erlenmeyers containing 75 ml of wort and incubated for 72 h at 20C and 160 rpm. Then, 50 ml was transferred to a 1000 ml Erlenmeyer with 400 ml of wort and incubated for 72 hs at 20°C under static conditions. The OD_600 nm_ was measured, and the appropriate amount of culture was centrifuged and resuspended in wort to reach an OD_600 nm_ of 0.5 in 450 ml wort in 500 ml Schott flasks. The flasks were shaken vigorously, closed with a plug and water lock and incubated for 7 days at 20°C (mimicking primary fermentation). After 7 days, the upper part of the ferment (250 ml) was transferred to beer bottles containing 3.5 ml of 50% sucrose solution (w/v) and locked with a cap (start secondary fermentation). These bottles were subsequently stored for 2 days at 20°C followed by incubation for 47 days at 4°C (end secondary fermentation).

The residual sugars and ethanol production (using HPLC), volatile organic compounds (HS‐SPME GC‐MS), pH, CO_2_ production (Δ weight of bottles) and viable counts were monitored. Two types of agar plates were used to obtain viable counts, a non‐selective plate for total viable counts and a selective plate to distinguish *C. fabianii* in co‐cultivation. MEA was used as non‐selective plate and incubated for 2 days at 30°C. The selective plate contained 1% sorbitol (w/v; SIGMA‐Aldrich Co., St. Louis, USA), 1× yeast nitrogen base w/o amino acids and ammonium (Becton, Dickinson and Company Sparks, USA), 45.4 mM (NH_4_)_2_SO_4_, 2% bacteriological agar (w/v; Oxoid Limited, Basingstoke, UK) and was incubated at 30°C for 2 days.

### Test effect ergosterol supplementation (Infors bioreactors)

The effect of ergosterol supplementation was investigated using Infors HT 500 ml bioreactors. The bioreactors contained 450 ml wort which was stirred at 400 rpm and aerated till complete saturation of the wort and headspace was obtained. The wort was supplemented with antifoam (0.1 ml antifoam A; SIGMA‐Aldrich Co., St. Louis, USA). Fermentation was executed in the absence or presence of 1% (v/v) 100× ET80. 100× ET80 consists of 2.5 mg ml^−1^ ergosterol (SIGMA‐Aldrich Co., St. Louis, USA) dissolved in ethanol (VWR Chemicals, Amsterdam, The Netherlands) and Tween 80 (Merck, KGaA, Darmstadt, Germany) (ratio 1:1). Prior to inoculation, the air inlet was closed and a water lock was attached to the air outlet. The bioreactors were stirred at 100 rpm to homogenize the wort, and a sample port was connected to take samples during the fermentation run. The wort (either with or without ET80) was inoculated with 10^4^ CFU ml^−1^ brewers’ yeast + 10^6^ CFU ml^−1^ *C. fabianii* as described in the section Wort fermentation in Schott flasks for co‐culture/dose–response experiment. The fermentation performance was studied for 7 days (equal to primary fermentation). During the fermentation, the pH, DO (dissolved oxygen), residual sugars and ethanol production, total viable plate counts, *C. fabianii* plate counts [see Materials & Methods wort fermentation in bottles (450 ml)] and the brewers’ yeast viable plate counts were determined using MEA plates supplemented with 1% ET80 (v/v, incubated anaerobically at 30°C for 2 days).

### Residual sugar and ethanol analysis (HPLC)

High‐performance liquid chromatography (HPLC) was performed to quantify ethanol, glucose, maltose and maltotriose on an Ultimate 3000 HPLC (Dionex) equipped with an RI‐101 refractive index detector (Shodex, Kawasaki, Japan), an autosampler and an ion‐exclusion Aminex HPX – 87H column (7.8 × 300 mm) with a guard column (Bio‐Rad, Hercules, CA). As mobile phase, 5 mM H_2_SO_4_ was used at a flow rate of 0.6 ml min^−1^ and the column was kept at 40°C. Total run‐time was 30 min. The injection volume was 10 μl. Samples were deproteinated with 0.5 volume Carrez A (0.1M potassium ferrocyanide trihydrate) and 0.5 volume Carrez B (0.2M zinc sulfate heptahydrate) and 2× diluted with Milli‐Q. Diluted samples and standards (4–20 mM for glucose, 8–40 mM for maltose and 2–10 mM for maltotriose; 0.72–3.6% (v/v) for ethanol) were injected onto the column.

### Volatile organic compound (VOC) analysis (using HS‐SPME GC‐MS)

The final beer was filtered (0.45 μm), and 2 ml was transferred to a GC‐MS vial. 10 μl of 0.4 mg ml^−1^ decane (dissolved in methanol) was added as an internal standard. Samples were kept frozen (−20°C) until analysis.

The following method was used to determine the volatile organic compounds present in the sample using headspace solid‐phase microextraction gas chromatography‐mass spectrometry (HS‐SPME GC‐MS) analysis.

Samples were defrosted and incubated for 5 min at 60°C, followed by extraction for 20 min at 60°C using a Solid‐Phase Microextraction (SPME) fiber (Car/DVB/PDMS, Supelco, Supelco Park, Bellefonte, PA, USA). The compounds were desorbed from the fibre for 10 min on a Stabilwax^®^‐DA‐Crossband^®^‐Carbowax^®^‐polyethylene‐glycol column (30 m length, 0.25 mm ID, 0.5 μm df). The gas chromatograph settings were PTV split‐less mode (5 min) at 250°C. Helium was used as carrier gas at a constant flow of 1.5 ml min^−1^. The GC oven temperature, initially at 40°C for 2 min, raised to 240°C (10°C min^−1^) and was kept at 240°C for 5 min. Total run‐time was 28 min. Mass spectral data were collected over a range of *m*/*z* 33–250 in full‐scan mode with 3.0030 scans s^−1^.

Peaks were annotated using Chromeleon^®^ 7.2. The ICIS algorithm was used for peak integration and the NIST main library to match the mass spectral profiles with the profiles of NIST. Peak areas were calculated using the MS quantification peak (highest *m*/*z* peak per compound).

For hierarchical clustering, the peak areas were normalized per compound using Log_2_(peak area/median (peak area all samples)). The hierarchical clustering was performed in MeV v4.8.1.

Shannon index was calculated to indicate the complexity of the compound composition. A higher index indicates a more complex aroma bouquet. The formula used was as follows:

$$\text{Shannon}\;\text{index}=-\sum \left(P_{i}\times Ln\left(P_{i}\right)\right)$$

$$P_{i}=\frac{\text{Peak area (TIC)}}{\Sigma \text{Peak}\;{\text{area}}^{\prime }s\left(\text{TIC}\right)}$$

### Statistics

An initial inoculum of 100% brewers’ yeast was used as reference. A Student's *t*‐test was used to determine whether other inoculation strategies resulted in a significantly different end‐product. A *P*‐value < 0.05 was used as cut‐off to indicate significance difference.

Only HS‐SPME GC‐MS data were first transferred to log_2_ values to obtain values that are equally distributed and have equal variance between the different treatments. After transformation of the values, Student's *t*‐test was used to test for significant differences (*P *<* *0.05).

## Supporting information


**Fig. S1.** brewers’ yeast growth yields on various filtrates to test if Cf65 produces inhibitory compounds against brewers’ yeast.
