# Supplementary figures and images for: Performance of non‐conventional yeasts in co‐culture with brewers’ yeast for steering ethanol and aroma production
Source: Microb Biotechnol. 2017 Aug 18;10(6):1591–602. doi: 10.1111/1751-7915.12717 (PMC5658577; doi:10.1111/1751-7915.12717)

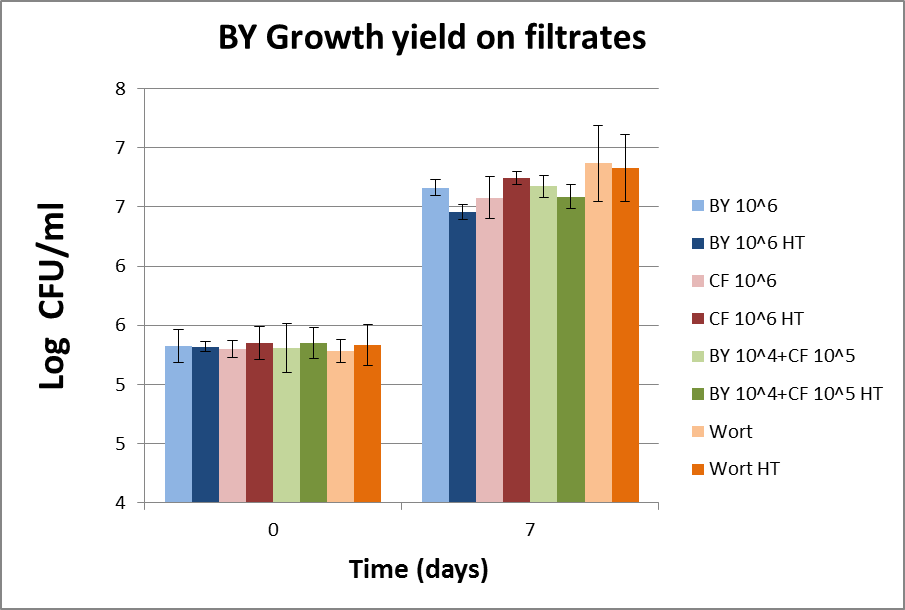

Supplement: Supplementary file 1 — Fig. S1. brewers’ yeast growth yields on various filtrates to test if Cf65 produces inhibitory compounds against brewers’ yeast. [file MBT2-10-1591-s001.tif]
